# Idiopathic acute myocarditis during treatment for controlled human malaria infection: a case report

**DOI:** 10.1186/1475-2875-13-38

**Published:** 2014-01-30

**Authors:** Maurits PA van Meer, Guido JH Bastiaens, Mohamed Boulaksil, Quirijn de Mast, Anusha Gunasekera, Stephen L Hoffman, Gheorghe Pop, André JAM van der Ven, Robert W Sauerwein

**Affiliations:** 1 Department of Medical Microbiology, Radboud University Medical Center, Nijmegen, The Netherlands; 2 Department of Cardiology, Radboud University Medical Center, Nijmegen, The Netherlands; 3 Department of General Internal Medicine, Radboud University Medical Center, Nijmegen, The Netherlands; 4 Sanaria Inc., Rockville, MD, USA

**Keywords:** CHMI, Myocarditis, Troponin T, MRI, Malaria

## Abstract

A 23-year-old healthy male volunteer took part in a clinical trial in which the volunteer took chloroquine chemoprophylaxis and received three intradermal doses at four-week intervals of aseptic, purified *Plasmodium falciparum* sporozoites to induce protective immunity against malaria. Fifty-nine days after the last administration of sporozoites and 32 days after the last dose of chloroquine the volunteer underwent controlled human malaria infection (CHMI) by the bites of five *P. falciparum*-infected mosquitoes. Eleven days post-CHMI a thick blood smear was positive (6 *P. falciparum*/μL blood) and treatment was initiated with atovaquone/proguanil (Malarone®). On the second day of treatment, day 12 post-CHMI, troponin T, a marker for cardiac tissue damage, began to rise above normal, and reached a maximum of 1,115 ng/L (upper range of normal = 14 ng/L) on day 16 post-CHMI. The volunteer had one ~20 minute episode of retrosternal chest pain and heavy feeling in his left arm on day 14 post-CHMI. ECG at the time revealed minor repolarization disturbances, and cardiac MRI demonstrated focal areas of subepicardial and midwall delayed enhancement of the left ventricle with some oedema and hypokinesia. A diagnosis of myocarditis was made. Troponin T levels were normal within 16 days and the volunteer recovered without clinical sequelae. Follow-up cardiac MRI at almost five months showed normal function of both ventricles and disappearance of oedema. Delayed enhancement of subepicardial and midwall regions decreased, but was still present. With the exception of a throat swab that was positive for rhinovirus on day 14 post-CHMI, no other tests for potential aetiologies of the myocarditis were positive. A number of possible aetiological factors may explain or have contributed to this case of myocarditis including, i) *P. falciparum* infection, ii) rhinovirus infection, iii) unidentified pathogens, iv) hyper-immunization (the volunteer received six travel vaccines between the last immunization and the CHMI), v) atovaquone/proguanil treatment, or vi) a combination of these factors. Definitive aetiology and pathophysiological mechanism for the myocarditis have not been established.

## Background

Controlled human malaria infections (CHMIs) have been used for nearly a century for treatment of neurosyphilis and for assessing interventions like drugs and vaccines for treating and preventing malaria. However, the modern era of CHMIs began in the mid 1980s, when laboratory reared *Anopheles* sp. mosquitoes were infected by feeding on cultures of *Plasmodium falciparum*-infected blood [1]. During the past three decades CHMI has been shown safe, well-tolerated and useful in evaluation of potential new anti-malarial drugs and vaccines [2]. After exposure to bites of laboratory-reared mosquitoes infected with *P. falciparum* sporozoites (PfSPZ) clinical symptoms and signs of malaria are generally mild to moderate and last for a few days. The most commonly reported symptoms are headache, fever, myalgia and fatigue, and common laboratory abnormalities include clinically insignificant thrombocytopaenia and leukopaenia [3,4]. Subjects, who undergo CHMI, are closely monitored and immediately treated with anti-malarials upon detection of parasitaemia. Due to frequent and intense clinical monitoring, initiation of treatment almost always occurs at parasite densities of less than 0.001% and often at 0.0001% [5], a density which is more than 1,000-fold lower than parasite densities associated with causing severe malaria.

Immunization of volunteers taking chloroquine chemoprophylaxis with whole PfSPZ administered by mosquito bites resulted in complete and long-lasting protection against CHMI with *P. falciparum*-infected mosquitoes [6,7]. This immunization approach is called “*C*hemo*P*rophylaxis with *S*porozoites” (CPS). Since CPS depends on inoculation of PfSPZ by mosquito bites, it cannot be an implementable vaccine. Recently, subjects were infected by needle and syringe inoculation of aseptic, purified, cryopreserved PfSPZ, a product called PfSPZ Challenge [8,9]. Subsequently, a clinical trial was initiated in which volunteers taking chloroquine chemoprophylaxis were injected intradermally (ID) at four-week interval with PfSPZ Challenge, an approach called the PfSPZ-CVac approach (=PfSPZ *C*hemoprophylaxis *Vac*cine), and then underwent CHMI.

Here, a very probable case of acute myocarditis is described in a volunteer who had taken chloroquine chemoprophylaxis, was inoculated three times at four-week intervals with PfSPZ Challenge, received six travel-related routine vaccines after this immunization procedure, had CHMI by the bites of five PfSPZ-infected mosquitoes 8.5 weeks after the last dose of PfSPZ Challenge and 4.5 weeks after the last dose of chloroquine, had a sore throat on day 9 after CHMI, developed *P. falciparum* parasitaemia that was treated 11 days after CHMI, and had asymptomatic initial elevation of troponin T levels 12 days after CHMI.

## Case presentation

A 23-year-old healthy male volunteer was enrolled in a double blind, randomized, controlled trial that assessed the safety, tolerability, and protective efficacy against CHMI by PfSPZ-infected mosquitoes of intradermal administration of aseptic purified cryopreserved PfSPZ (PfSPZ Challenge) in volunteers taking weekly 300 mg chloroquine prophylaxis, the PfSPZ-CVac approach. His medical history was unremarkable, and he did not smoke or use illicit drugs. His mother had a history of hypertension and his paternal grandfather had a history of heart valve defects and a possible myocardial infarction at the age of 70. At inclusion, physical examination was within normal limits with a blood pressure of 139/76 mmHg, heart rate of 55 beats per minute and a body mass index of 20.2 kg/m^2^. Electrocardiography (ECG) showed a commonly seen normal variant of incomplete right bundle branch block (see Additional file 1). Standard laboratory tests at inclusion were normal (see Table 1).

**Table 1:** Laboratory findings

**Haematology and biochemistry tests**	**Normal range**	**Inclusion**	**C +11**	**C +12**	**C +13**	**C +14**	**C +15**	**C +16**	**C +17**	**C +20**	**C +28**
Haemoglobin (mmol/L)	8.5 – 10.8	10.1	9.4	10.2	9.2	8.2	8.2	8.2	8.0	9.5	9.6
Haematocrit (L/L)	0.41 – 0.53	0.47	0.43	0.47	0.43	0.38	0.37	0.38	0.37	0.44	0.44
Leukocytes (x10^9^/L)	3.6 – 10.7	4.9	4.2	3.3	3.2	3.1	3.9	4.0	4.8	7.7	5.4
Neutrophils (x10^9^/L)	2.0 – 7.5	2.16	3.04	1.85	1.77	1.26					2.12
Lymphocytes (x10^9^/L)	1.0 – 3.5	2.05	0.60	0.79	0.88	1.13					2.57
Monocytes (x10^9^/L)	0.3 – 1.0	0.43	0.48	0.52	0.47	0.55					0.47
Eosinophils (x10^9^/L)	≤ 0.64	0.21	0.08	0.08	0.10	0.09					0.21
Basophils (x10^9^/L)	< 0.10	0.03	0.03	0.02	0.02	0.03					0.05
Thrombocytes (x10^9^/L)	141 – 400	138	97	94	84	89	108	123	129	200	162
Sodium (mmol/L)	135 – 143	139			138					142	
Potassium (mmol/L)	3.7 – 5.0	3.8			4.0					3.8	
Creatinine (μmol/L)	60 – 132	87			82	74	83	80	76	75	
Urea nitrogen (mmol/L)	2.5 – 8.1	5.1			4.6					5.8	
Alkaline phosphatase (U/L)	≤ 126	66			82	84	76	77	76		
Aspartate aminotransferase (U/L)	≤ 38	16			49	81	66	41	32		
Alanine aminotransferase (U/L)	≤ 49	23			33	42	37	41	40		
Lactate dehydrogenase (U/L)	≤ 250	110	142	181	222	316	262	258	222		154
γ Glutamyl-transferase (U/L)	≤ 55	22			18	21	19	23	22		
Creatine Kinase (U/L)	≤ 170			175	284	501	320	133	70		
Troponin T (ng/L)	≤ 14	6	8	45	197	596	829	1115	675	18	7
NT-proBNP (pg/mL)	< 88						197		128	67	
CRP (mg/L)	≤ 10				22	16	11	8	< 5	< 5	
D-dimer (ng/mL)	≤ 500	< 500	< 500	570	< 500	< 500	< 500	< 500	< 500		< 500

Clinical laboratory findings at inclusion (day before the start of the trial), on day of thick smear positivity (C + 11, day 11 after CHMI) and subsequent days 12, 13, 14, 15, 16, 17, 20, and 28 after CHMI.

From October to December 2012, he received three intradermal injections at four-week intervals of 7.5 ×10^4^ PfSPZ of PfSPZ Challenge (PfNF54) diluted in phosphate buffered saline with 1% human serum albumin. From day 3 until day 8 after the first immunization he reported a sore throat and symptoms of a common cold (i.e., stuffy nose and coughing) with mild chills for a few hours. No complaints were reported after the second and third immunizations. No clinically significant laboratory abnormalities were found during the immunization period.

Fifty-nine days after the third and last immunization and 32 days after his last dose of chloroquine, he underwent CHMI by the bites of five *P. falciparum*-infected mosquitoes (PfNF54). On day 9 post-CHMI he complained of a sore throat. On day 11 post-CHMI his thick blood smear became positive (6 *P. falciparum*/μL blood; 0.00012% infected erythrocytes) and standard treatment with Malarone® (1,000 mg atovaquone plus 400 mg proguanil once daily for three days) was initiated. Retrospective assessment of parasitaemia by quantitative polymerase chain reaction (qPCR) revealed 13,293 parasites/mL (0.00026% infected erythrocytes) on day of thick smear positivity. On that day he complained of minor chills and headache for a few hours with a highest recorded sublingual temperature of 37.5°C. Platelet and lymphocyte counts decreased to 97 ×10^9^/L (normal range = 141 – 400 ×10^9^/L) and 0.60 ×10^9^/L (normal range = 1.0 – 3.5 ×10^9^/L), respectively, as often seen in malaria positive individuals [5,10]. The level of troponin T by a highly sensitive assay was normal (i.e., 8 ng/L; upper limit of normal = 14 ng/L). Troponin T is a specific marker for myocardial tissue damage.

On the second day of Malarone treatment (day 12 post-CHMI) the troponin T level was elevated at 45 ng/L and increased to 63 ng/L in the evening. No abnormalities were seen on ECG. Apart from mild headache and fatigue on the following day (day 13 post-CHMI) the volunteer was asymptomatic, but the troponin T was 197 ng/L in the morning and 299 ng/L in the afternoon. The blood pressure was 114/60 mmHg and the ECG revealed mild repolarization disturbances with diffuse ST-T-segment elevation, suggestive of pericarditis (see Additional file 2). The echocardiogram showed mild hypokinesia of the inferior wall and a slightly diminished global left ventricle (LV)-function (calculated LV ejection fraction of 53%; normal range for a young man is > 55%). Malarone treatment was completed and qPCR for *P. falciparum* was negative on day 13 post-CHMI. Although the subject did not have any cardiac or chest symptoms, he was hospitalized at the cardiology department according to safety protocol for telemetric ECG-monitoring and follow-up of troponin T levels.

**Figure 1:**
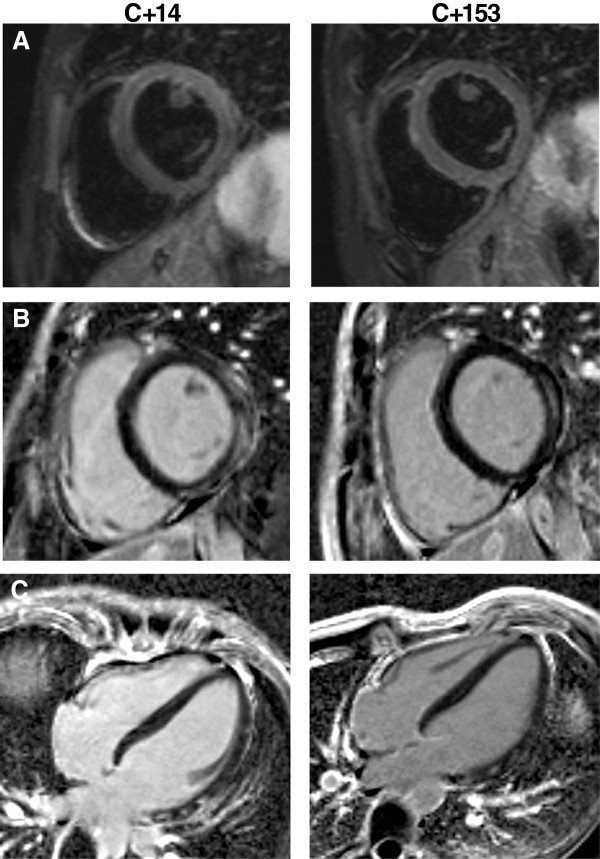
**Cardiac MRI on day 14 and 153 after CHMI. (A)** Slightly increased T2-weighted signal intensity was observed in the basal-inferolateral segment of the left ventricle on day 14 after CHMI (C + 14), which had disappeared on day 153 after CHMI (C + 153); visualized on the short-axis dark blood STIR (short inversion time inversion recovery) recordings. **(B and C)** After administration of 15 mL gadolinium contrast subepicardial and midwall delayed enhancement was observed in the basal-inferolateral and basal-inferior segments of the left ventricle on day C + 14, which had decreased on day C + 153; visualized on the short-axis **(B)** and the 4-chamber **(C)** PSIR (phase sensitive inversion recovery) recordings.

That night at ~01:00 AM he experienced retrosternal pain and a heavy feeling in his left arm. After approximately 10 minutes sublingual nitroglycerin spray was administered; the chest pain did not disappear immediately, but only 10 minutes after administration of nitroglycerin. The pain was not related to breathing and there were no concomitant complaints or signs of dyspnoea, pyrosis or ructus. The subject never had another episode of chest pain. Cardiac MRI several hours later on day 14 post-CHMI showed: i) slightly increased T2-weighted signal intensity in the basal- and mid-inferolateral and partly in the mid-anterolateral myocardial segments, matching minor oedema (see Figure 1A); ii) focal areas of subepicardial and midwall delayed enhancement in the basal- and mid-inferior and basal- and mid-inferolateral segments after administration of gadolinium contrast (see Figure 1B and C); iii) hypokinesia basal- and mid-inferior and mild hypokinesia basal- and mid-inferolateral. These findings were interpreted as indicative of myocarditis. Treatment was started on day 14 post-CHMI with a beta-blocker, metoprolol (25 mg twice daily), to reduce the chance of cardiac arrhythmia and according to the treatment guidelines for patients with reduced LV-function.

Troponin T levels continued to rise with a peak of 1,115 ng/L on day 16 after CHMI. The following days troponin T decreased and eventually returned to normal 16 days after initial increase, corresponding to 28 days post-CHMI. Creatine kinase (CK) showed a similar pattern of rising and falling, but returned to normal on day 16 post-CHMI. A biochemical marker of cardiac wall stress, N-terminal pro-hormone brain natriuretic peptide (NT-proBNP), was slightly elevated on days 15 and 17 post-CHMI, but was normal on day 20 post-CHMI (see Table 1). A limited rise and fall of aspartate aminotransferase (AST) and lactate dehydrogenase (LDH) were found. The nonspecific marker for increased coagulation and inflammation, D-dimer, remained within the normal range and was only minimally elevated on the first day after thick smear positivity. Similarly, the inflammatory acute-phase protein, C-reactive protein (CRP) was only slightly elevated on days 13, 14, and 15 after CHMI (see Table 1). Four days after admission (day 17 post-CHMI) ECG and echocardiogram had normalized (see Additional file 3 and calculated LV ejection fraction was 62%; normal range is > 55%) and the volunteer was discharged.

Apart from the single short episode of chest pain and a longer period of fatigue with occasional mild headache during and shortly after hospitalization, no other complaints were reported. The fatigue diminished after halving the dose of metoprolol to 25 mg once daily on day 23 post-CHMI. The remaining mild fatigue completely disappeared three weeks later on day 44 post-CHMI. After discharge the volunteer did not complain about occasional mild headache anymore.

The volunteer received pre-travel vaccines for diphtheria, tetanus, polio, typhus, hepatitis A and hepatitis B 14 days after the third injection of PfSPZ Challenge (46 days before CHMI). He had booster vaccinations for hepatitis A and B 40 days after the third injection (20 days before CHMI).

Polymerase chain reaction (PCR) analyses of throat smear, faeces and whole blood were carried out for viruses and bacteria known to cause myocarditis (see Table 2). Throat smear PCR was positive for rhinovirus; all other PCR results were negative (Table 2). Virological, bacteriological and parasitological serology was performed on samples obtained three weeks before inclusion and on day 17 post-CHMI (see Table 3) with repeat testing 18 days later (day 35 post-CHMI and 23 days after first elevation of troponin T). All serologic results were negative. Furthermore, urine toxicology screening for amphetamine-derivatives, cocaine, cannabinoids, diazepam, methadone, tramadol hydrochloride, and opiates was negative on day 17 after CHMI.

**Table 2:** PCR results for known infectious pathogens of myocarditis

	**C + 14**	**C + 15**	**C + 17**
**Throat smear tested by PCR for:**			
Adenovirus, Bocavirus, Coronavirus, Chlamydia Psittaci, Enteroviruses, Metapneumovirus, Mycoplasma, Parechovirus, Parainfluenza 1 – 4, Rhinovirus, Respiratory Syncytial Virus, and Influenza A and B	positive for Rhinovirus		
**Faeces tested by PCR for:**			
Adenovirus (Adenotype 40 and 41), Astrovirus, Bocavirus, Enteroviruses, Norovirus, Parechovirus, Rotavirus, and Sapovirus		negative	
**Blood tested by PCR for:**			
Varicella Zoster Virus, Parvovirus, Epstein-Barr Virus, Cytomegalovirus, Q fever, and HIV load			negative

PCR results for known virological and bacteriological causes of myocarditis based on samples taken on day 14, 15, and 17 after CHMI (C + 14, C + 15, and C + 17, respectively).

**Table 3:** Serology results for known infectious pathogens of myocarditis

**Sera tested for antibodies to:**	**Screening visit (paired with C + 17)**	**C + 17**	**C + 17 (paired with C + 35)**	**C + 35**
Echovirus pool	20	< 10 (negative)	< 10 (negative)	10
(Types 4, 6, 9, 14, 24, and 30)				
Coxsackie virus pool	20	10	10	20
(Types A9, B1 – B6 )				
Poliovirus	20	10	20	40
Adenovirus Ig	20	< 10 (negative)	20	20
Parvovirus IgM		negative		negative
Parvovirus IgG		64		64
Mycoplasma pneumoniae IgM	negative	negative		negative
Mycoplasma pneumoniae IgA	negative	negative		negative
Mycoplasma pneumoniae Ig	< 10 (negative)	< 10 (negative)	< 10 (negative)	< 10 (negative)
Chlamydia including Psittacosis Ig	< 10 (negative)	< 10 (negative)	< 10 (negative)	< 10 (negative)
Q fever phase 2 IgM	negative	negative		negative
Q fever Ig	< 10 (negative)	< 10 (negative)	< 10 (negative)	< 10 (negative)
Hepatitis B s-antigen		negative		negative
Hepatitis B anti-HBc		negative		negative
Hepatitis C Ig		negative		negative
Borrelia blot IgM		negative		negative
Borrelia blot IgG		negative		negative
Legionella serotype 1 – 7 IgM	negative	negative		negative
Legionella serotype 1 – 7 IgG	negative	negative		negative
Syphilis IgG		negative		negative
Toxoplasma IgG		negative		negative

Serology results for known virological, bacteriological and parasitological causes of myocarditis based on samples taken at the screening visit, on day 17, and 35 after CHMI (C + 17 and C + 35, respectively). Paired serologic analysis was performed with serum drawn at the screening visit (three weeks before the start of the trial) for a number of pathogens. To detect potential delayed immune responses serologic analysis was repeated on day C + 35. Again, paired serologic analysis was performed with serum drawn on day C + 17 for a number of pathogens.

At almost five months after the first MRI, repeat cardiac MRI demonstrated good function of the left ventricle (calculated LV ejection fraction of 67%) with persistence of mild hypokinesia in the mid-inferior and mid-inferolateral segments of the left ventricle. The oedema had disappeared completely (see Figure 1A) and concomitantly the delayed enhancement had decreased, mostly in the basal-inferolateral segment (see Figure 1B and C). However, patchy midwall and subepicardial delayed enhancement was still present in four myocardial segments (i.e., the basal-inferolateral, mid-inferolateral, basal-inferior, and mid-inferior segments). Follow-up ECG did not show any abnormalities except for a minimally widened QRS complex compared to the pre-trial ECG and the persistence of incomplete right bundle branch block (see Additional file 4). In addition, ECG during a cardiac stress test did not show ST-T-segment changes, rhythm abnormalities, or other changes with respect to the pre-trial ECG. The cardiac stress test used a cycling protocol starting at 50 watt and with increasing steps of 20 watt per minute. He reached a maximally achieved power of 270 watt (i.e., 122% of expected for his age group and gender). He had an adequate increase in blood pressure and heart rate. His heart rate pressure product was 35,854 mmHg/min (normal is > 25,000 mmHg/min). Metoprolol 25 mg once daily was stopped and the volunteer has remained without complaints in good condition.

## Discussion

Clinically suspected acute myocarditis with typical MRI characteristics is reported in a healthy volunteer participating in a PfSPZ-CVac approach phase 1 clinical trial. The first myocarditis manifestations occurred 71 days after the last dose of PfSPZ Challenge, 44 days after the last dose of chloroquine, 57 and 31 days after receiving pre-travel vaccines, 12 days after CHMI by *P. falciparum*-infected mosquito bites, three days after the onset of a sore throat, and one day after diagnosis of *P. falciparum* malaria and initiation of treatment for malaria. The retrosternal chest pain [11], kinetics of increased troponin T plasma concentrations, ECG and echocardiogram findings, and MRI findings, which are consistent with the guidelines of the International Consensus Group on MR Diagnosis of Myocarditis [12], support the diagnosis of acute myocarditis. Moreover, improved myocardial function, disappearance of oedema and reduced delayed enhancement after almost five months correspond to the natural course of acute myocarditis; the residual delayed enhancement is consistent with contrast retention in fibrous tissue [13]. In addition, BNP and NT-proBNP were temporarily elevated and their elevation has also been found in patients with myocarditis and is associated with reduced left ventricular function [14,15].

The occurrence of the cardiac event relates in time with residual parasitaemia during curative Malarone treatment post-CHMI that might be suggestive of a causal relationship. A few cases of malaria and concomitant myocarditis have been reported in the literature, albeit restricted to patients with severe or fatal infection with *P. falciparum *[16-22] and *Plasmodium vivax* malaria [23]. In literature, myocarditis has never been reported in patients with uncomplicated *P. falciparum* malaria, even in those patients who present with *P. falciparum* parasite densities 20 to 30 times higher than the parasite density in this volunteer. In addition, troponin T plasma concentrations have never shown elevations above background in an unselected group of 167 volunteers when daily measured after CHMI using a highly sensitive assay (personal communication by RW Sauerwein (Radboud university medical center, The Netherlands)). Consistent with this finding, troponin T was very rarely (0.6%) elevated when assessed retrospectively in patients with uncomplicated *P. falciparum* malaria [24]. In contrast, 31 – 80.5% of African children with severe and/or fatal *P. falciparum* malaria exhibited high to very high levels of circulating cardiac proteins indicating myocardial injury and impaired left ventricular function [25].

Previously, a cardiac serious adverse event was reported in a female volunteer who participated in a phase 1 clinical trial in which she was immunized with a subunit, recombinant protein malaria vaccine (PfLSA3), underwent CHMI by mosquito bites, developed malaria, and was treated with Riamet® (artemether/lumefantrine) [26]. She was diagnosed with acute coronary syndrome two days after completing treatment, but myocarditis was considered a possible alternative diagnosis. Apart from one confirmed myocardial infarction in a male volunteer with an increased cardiovascular risk, who underwent CHMI but did not develop parasitaemia [3], there have been no other reports of cardiac complications in the approximately 2,000 subjects who have undergone CHMI since the 1986 report by Chulay *et al. *[1].

In the current case the cardiac event occurred during the three days when the subject was receiving curative Malarone treatment for *P. falciparum* malaria. There are no previous data indicating that anti-malarial treatment with atovaquone/proguanil (Malarone®), or its metabolite cycloguanil causes myocarditis or any other significant cardiovascular toxicity [27]. Furthermore, the product monograph of Malarone does not mention cardiotoxicity or myocarditis, only palpitations and tachycardia.

The most common cause of acute myocarditis in a healthy young individual is a viral infection [28]. Numerous infectious pathogens can cause acute myocarditis [29,30]. Enteroviridae (including Coxsackie B) were responsible for 25 – 30% of cases in the past [31], but more recently, other viruses (including adenovirus, parvovirus B19, and hepatitis C) have also emerged as important cardiotropic pathogens [32]*.* In the current volunteer, diagnostic tests for the most common infectious causes of myocarditis were negative (see Table 2 and 3). However, negative convalescent antibody titers do not exclude a post-infectious myocarditis. Furthermore, a throat swab, taken two days after the initial increase in troponin T contained rhinovirus, and rhinovirus has been occasionally associated with myocarditis [33,34]. Noteworthy, rhinovirus is often detected by PCR in asymptomatic subjects and a causal inference with symptomatic patients should, therefore, be made with caution [35].

The pathogenesis of myocarditis can be due to direct infection of the myocardium by a replicating pathogen, the host’s specific immunologic response to such an infection [36], or a nonspecific immunologic response in a susceptible individual that could have been triggered in this case by the malaria infection. In most such cases one would expect to find markers of inflammation elevated. However, markers for nonspecific inflammation and haemolysis, D-dimer, CRP, and LDH, were normal or only slightly elevated when the troponin T levels were highest (see Table 1). Nonetheless, it is possible that an overall hyperreactivity induced by the six standard vaccines (i.e., diphtheria, poliomyelitis, tetanus, parenteral typhoid fever, hepatitis A and hepatitis B) the volunteer received between the immunization period and CHMI, and the subsequent CHMI could have generated a hypersensitivity myocarditis. Such post-vaccination myocarditis has been rarely reported for the administered vaccines and usually manifests with fever and/or other nonspecific inflammatory symptoms within several days of the hyperimmunizations [37], which did not occur in this volunteer. Nonetheless, this explanation cannot be ruled out.

No systemic allergic reactions or local adverse events have occurred in the 20 volunteers, who have now received three intradermal (ID) injections of 7.5 ×10^4^ PfSPZ of PfSPZ Challenge. Moreover, there have been no systemic allergic reactions among the 184 subjects who have received single doses of PfSPZ Challenge by the ID (n = 84), intramuscular (IM) (n = 70), and intravenous (IV) (n = 30) routes in order to study the safety and infectivity of PfSPZ Challenge ([8,9] and personal communication by SL Hoffman (Sanaria Inc., USA)), or among the 120 volunteers who have received up to six doses of 1.35 ×10^5^ radiation-attenuated PfSPZ (PfSPZ Vaccine) ID (n = 40), subcutaneously (SC) (n = 40), or IV (n = 40) [38,39]. Thus, it seems extremely unlikely that the parenterally administered PfSPZ or the phosphate buffered saline or human serum albumin with which the PfSPZ are administered contributed to the myocarditis or contain an immunologically sensitizing agent.

Myocarditis may also be triggered by toxins, alcohol, cocaine, chemotherapeutics, antibiotics, metabolic abnormalities, and other factors [29,30]. However, the volunteer denied excessive use of alcohol, and urine drug tests for cocaine, amphetamines and cannabinoids were negative, making such factors an unlikely explanation. Alcohol intake was not quantified during follow-up visits after CHMI, but volunteers were repetitively instructed to restrict alcohol intake. Since the urine drug test was performed five days after the first rise in troponin T, the detection of metabolites of cocaine and amphetamines after single use is quite limited at this time and could have been missed [40].

In conclusion, there are different possible causes for the myocarditis but a definitive cause in this case cannot be established. It is also possible that a combination of the above-discussed potential aetiological factors could have contributed to the development of this case of acute myocarditis.

## Consent

Informed consent for publication of this case report was obtained from the volunteer who participated in this clinical trial.

## Competing interests

The authors declare that they have no competing interests. However, SLH and AG are employees of Sanaria Inc., the manufacturer of PfSPZ Challenge.

## Authors’ contributions

MPAvM, GJHB, QdM, and AJAMvdV were clinical investigators. GP and MB were consultants in cardiology. SLH initiated and coordinated the clinical trial. AG carried out the regulatory affairs of the clinical trial. RWS was principal investigator. MPAvM, GJHB, and RWS wrote the paper with comments from the other authors. All authors read and approved the manuscript.

## Supplementary Material

Additional file 1 Electrocardiogram at screening visit before start of the clinical trial (10-SEP-2012, 10:59 AM) showing a normal variant of an incomplete right bundle branch block. Click here for file

Additional file 2 Electrocardiogram on day 13 after CHMI (18-FEB-2013, 07:41 PM) showing mild repolarization disturbances with diffuse ST-T-segment elevation. Click here for file

Additional file 3 Electrocardiogram on day 16 after CHMI (21-FEB-2013, 12:13 PM) showing normalization of the repolarization disturbances compared to the previous ECG of 18-FEB-2013. Click here for file

Additional file 4 Electrocardiogram on day 153 after CHMI (08-JUL-2013, 09:48 AM) showing no abnormalities except for the known incomplete right bundle branch block and a minimally widened QRS complex compared to the pre-trial ECG. Click here for file
