# Supplementary figures and images for: Idiopathic acute myocarditis during treatment for controlled human malaria infection: a case report
Source: Malar J. 2014 Jan 30;13:38. doi: 10.1186/1475-2875-13-38 (PMC3909449; doi:10.1186/1475-2875-13-38)

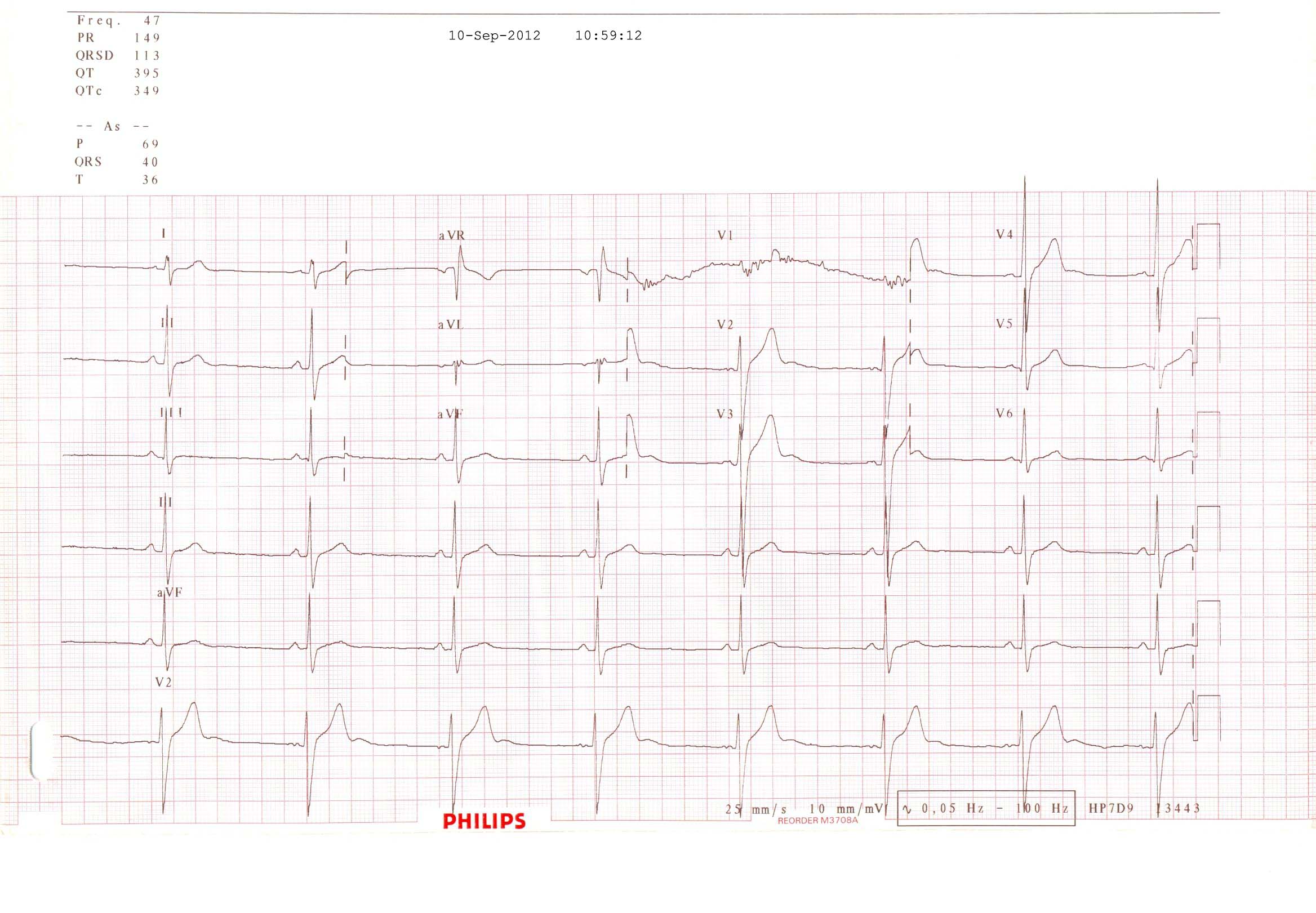

Supplement: Additional file 1 — Electrocardiogram at screening visit before start of the clinical trial (10-SEP-2012, 10:59 AM) showing a normal variant of an incomplete right bundle branch block. [file 1475-2875-13-38-S1.jpeg]

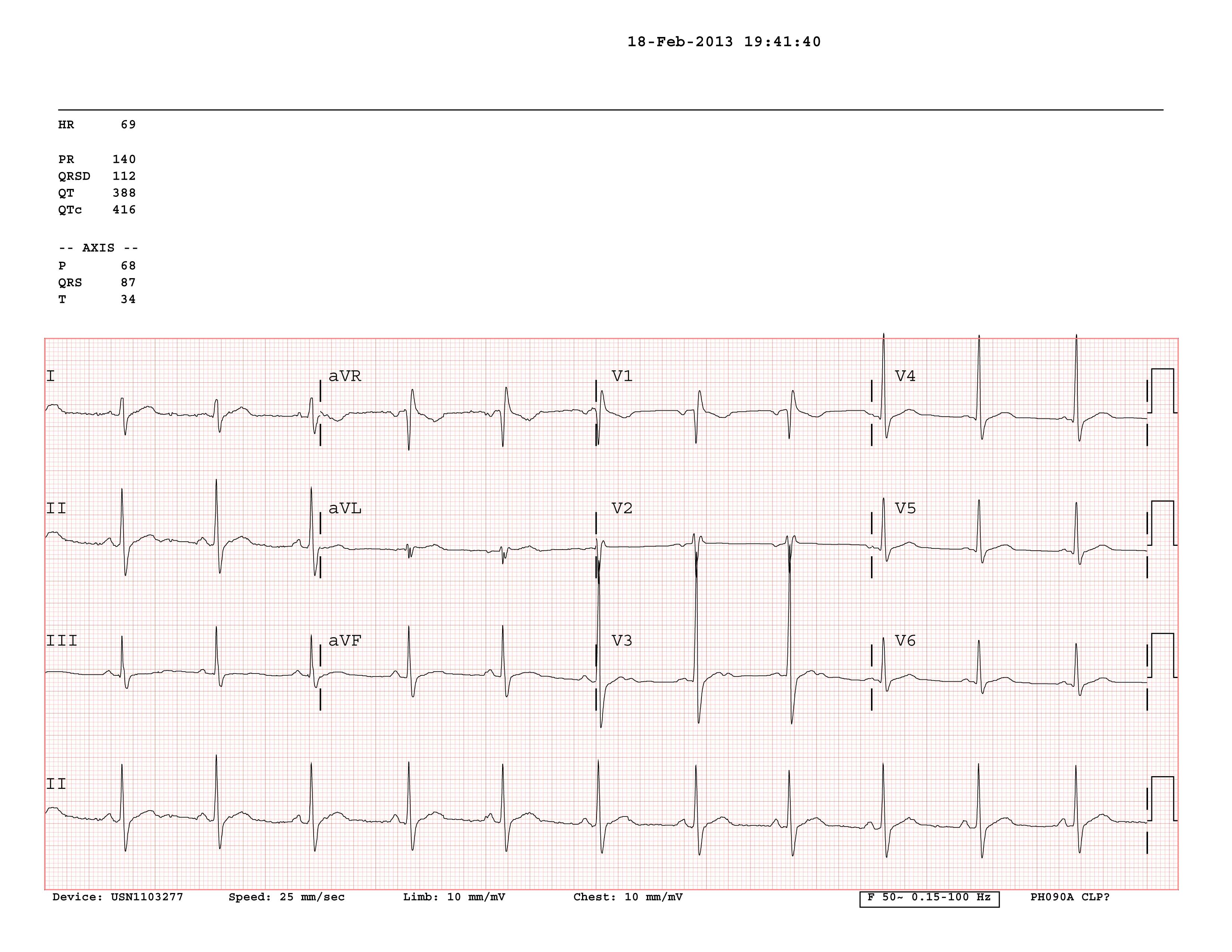

Supplement: Additional file 2 — Electrocardiogram on day 13 after CHMI (18-FEB-2013, 07:41 PM) showing mild repolarization disturbances with diffuse ST-T-segment elevation. [file 1475-2875-13-38-S2.jpeg]

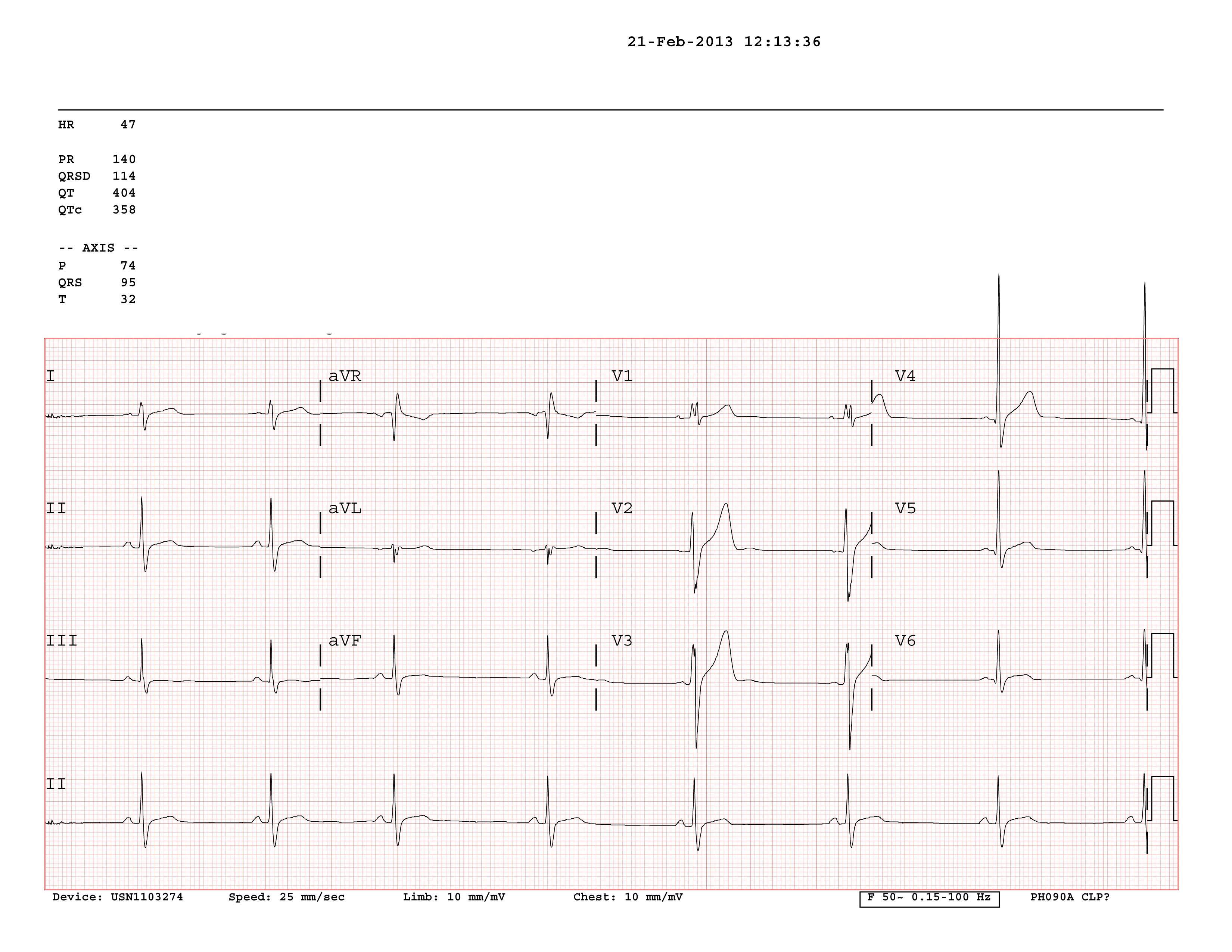

Supplement: Additional file 3 — Electrocardiogram on day 16 after CHMI (21-FEB-2013, 12:13 PM) showing normalization of the repolarization disturbances compared to the previous ECG of 18-FEB-2013. [file 1475-2875-13-38-S3.jpeg]

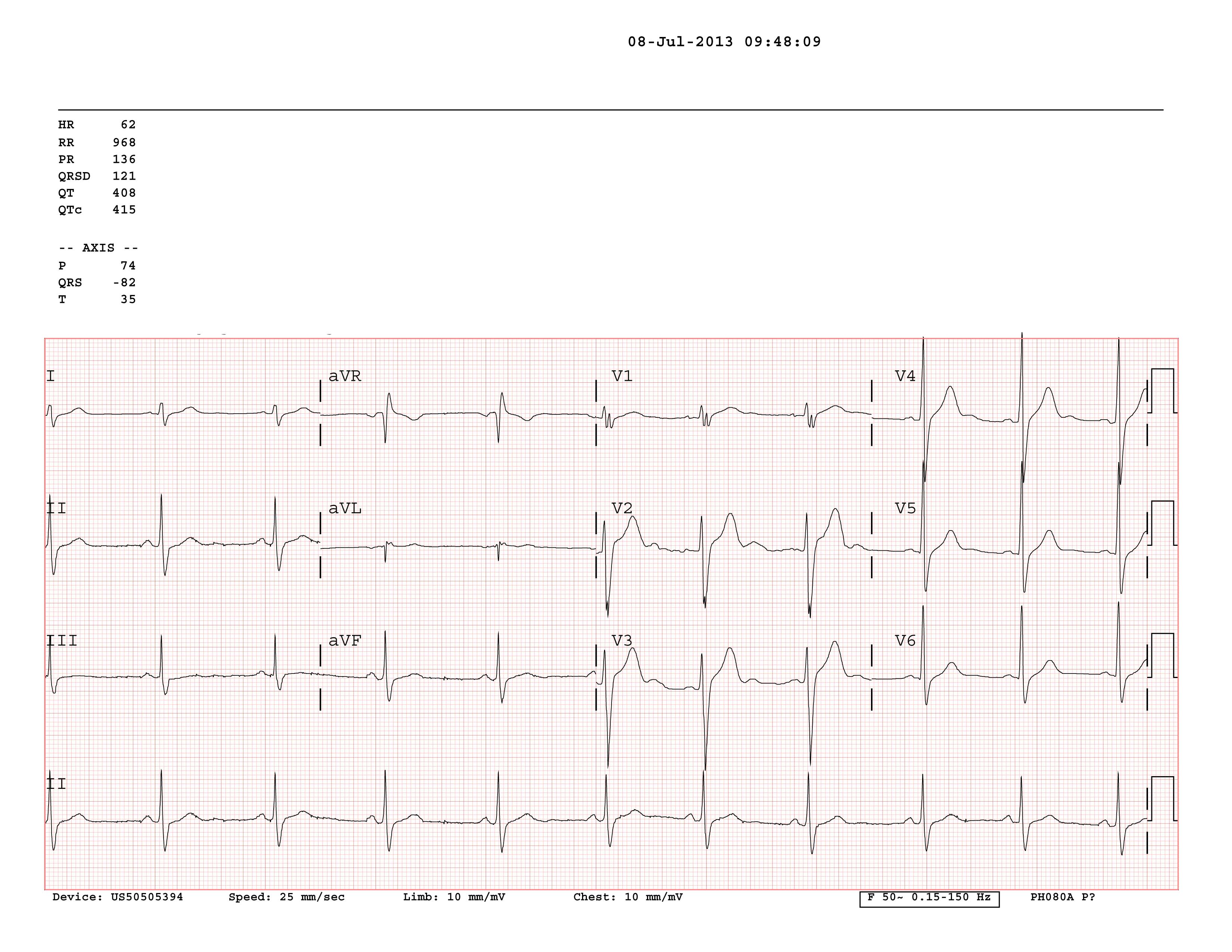

Supplement: Additional file 4 — Electrocardiogram on day 153 after CHMI (08-JUL-2013, 09:48 AM) showing no abnormalities except for the known incomplete right bundle branch block and a minimally widened QRS complex compared to the pre-trial ECG. [file 1475-2875-13-38-S4.jpeg]
